# No-Test Screening Protocols May Disproportionately Exclude Structurally Oppressed Communities Who Could Benefit from Accessing Medication Abortion

**DOI:** 10.1089/heq.2024.0128

**Published:** 2025-03-26

**Authors:** M. Antonia Biggs, Lauren Ralph, Katherine Ehrenreich, Shelly Kaller, Kelly Blanchard, Deb Hauser, Nathalie Kapp, Tammi Kromenaker, Ghazaleh Moayedi, Jamila Perritt, Elizabeth Raymond, Kari White, Daniel Grossman

**Affiliations:** ^1^ Advancing New Standards in Reproductive Health (ANSIRH), Bixby Center for Global Reproductive Health, Department of Obstetrics, Gynecology and Reproductive Sciences, School of Medicine, University of California San Francisco, Oakland, California, USA; ^2^ Ibis Reproductive Health, Cambridge, Massachusetts, USA.; ^3^ Advocates for Youth, Washington, District of Columbia, USA.; ^4^ International Planned Parenthood Federation, London, United Kingdom.; ^5^ Red River Women’s Clinic, Moorhead, Minnesota, USA.; ^6^ Pegasus Health Justice Center, Dallas, Texas, USA.; ^7^ Physicians for Reproductive Health, Washington, District of Columbia, USA.; ^8^ University of Washington, Tacoma, Washington, USA.; ^9^ Resound Research for Reproductive Health, Austin, Texas, USA.

**Keywords:** abortion, pregnancy, no-test, health screening

## Abstract

**Objective:**

To explore disparities in ineligibility for medication abortion using no-test screening criteria compared with assessment with testing including ultrasound.

**Methods:**

From June 2021 to December 2022, we surveyed patients ages ≥15 seeking abortion at nine recruitment facilities in eight U.S. states before ultrasound; clinicians assessed patients’ eligibility for medication abortion with ultrasound and other testing. Using clinical guidelines and the no-test protocol criteria, we estimated (1) the proportion ineligible by no-test assessment (pregnancy duration and ectopic pregnancy risk factors as reported in patient surveys and clinician-reported assessment of other contraindications) and (2) the proportion ineligible using no-test criteria yet eligible with testing (false positives). We assessed associations between participant characteristics and ineligibility for medication abortion and reasons for ineligibility.

**Results:**

We approached 2,846 people, of whom 1,591 were eligible for the study. Of the 1,386 who consented and had complete clinician data, 21.1% (306/1,386) were ineligible with testing, 71.5% (*n* = 991/1,386) were ineligible using no-test criteria, and 51.4% (*n* = 713/1,386) screened false positive. In adjusted analyses, ineligibility using no-test criteria was significantly greater among people ages 15–19 (86.8% [105/121] vs. 71.5% for full sample, *p* < 0.001) and experiencing food or housing insecurity (75.8% [525/690] vs. 67.2%[464/693], *p* < 0.01); people ages 20–24 were more likely to screen false positive (56.1% [263/469] vs. 51.4% for full sample [713/1,386], *p* = 0.03). Moderate/severe pelvic pain was the most common (614/1,386) patient-reported reason for ineligibility and reported significantly more by people ages 15–19, who were nulliparous, and experienced food or housing insecurity.

**Conclusions:**

Screening criteria for no-test medication abortion may exclude many people who are eligible, disproportionately excluding certain population groups from getting the care they seek. More research is needed to improve screening criteria to ensure equitable access to no-test medication abortion.

## Introduction

No-test screening for eligibility of medication abortion has emerged as an innovative person-centered approach to expand access to abortion by eliminating pre-abortion screening tests such as ultrasound, pelvic exam, and laboratory tests.^1,2^ A published sample no-test protocol provides screening criteria that exclude patients who report more than 77 days gestation or are unsure, who report unusual bleeding (vaginal bleeding or spotting) or pain symptoms (significant unilateral or bilateral pelvic pain) within the past week, and who have a history of or current ectopic pregnancy, surgery to the fallopian tubes, or an intrauterine device (IUD) in place, among other contraindications (hemorrhagic disorder, chronic adrenal failure, use of corticosteroid therapy, inherited porphyria, and allergy to mifepristone, or misoprostol).^1^ No-test screening can occur in the context of an in-person or telehealth visit where people interact with a clinician online, via text messaging, or telephone and receive the medication abortion regimen (mifepristone and misoprostol medications) by mail. People assessed ineligible for no-test medication abortion are typically referred to in-person testing and evaluation to confirm their eligibility for medication abortion. The no-test protocol has been widely adopted by abortion providers throughout the United States and globally, and research has demonstrated it to be as safe and effective as care that requires ultrasound and other pre-abortion screening tests.^3–9^ Most patients indicate a future preference for no-test screening and high levels of satisfaction.^6,7,10,11^

In the United States, with the removal of the federal protection of the right to abortion, no-test screening has become essential to meeting some of the demand for abortion, in particular for people living in states that ban or restrict access to abortion.^12,13^ “Shield laws” aim to provide additional legal protections to clinicians in states with protected access to abortion, enabling some clinicians to provide telehealth services using the no-test protocol and mailing the medications to patients anywhere in the United States.^14,15^

For this study, we consider health equity in the context of abortion care as rooted in reproductive justice principals^16^ and as ensuring that everyone who desires or needs an abortion can have a safe and timely abortion, of their preferred method and care model, of high quality, with dignity, respect and without judgment, if and when they choose. Health equity can only be achieved by eliminating health disparities, with access to health care considered one of the important social determinants of health.^17,18^ The underlying goal of the no-test approach is to reduce barriers and increase access to abortion care, which strongly aligns with these health equity goals. Research examining whether the no-test screening approach helps to improve equitable access to abortion and for whom it is accessible or preferable is sparse. One geospatial analysis found that telehealth, no-test medication abortion services reduced lengthy travel distances, in particular for young people, people experiencing food insecurity, and people later in pregnancy.^19^ Another study of 1,689 patients at one clinic in Washington State found that people living in rural areas were significantly more likely to access telehealth abortion care. However, they also pointed to potential disparities in access and/or preferences, with young people, Black people, and people who used interpreter services during their appointment being less likely to present for care via telehealth than in-person when compared to their counterparts.^20^ That same study reported that 26.5% of the sample was ineligible for telehealth services because during telephone screening, patients reported irregular periods (13%), pelvic pain (8%), gestational duration >70 days (2.5%), and an IUD in place (*n* = 1); the patient characteristics of those ineligible were not reported.

No-test screening may disproportionately exclude certain groups of people from no-test medication abortion. Some groups may be more likely to have certain contraindications or symptoms suggesting possible ectopic pregnancy or anemia, to lack access to home pregnancy tests and adequate health care to promptly confirm pregnancy duration and to know whether they have any medical contraindications for medication abortion.^21–25^ However, we lack empirical research on the characteristics of people who may be unable to access no-test medication abortion using no-test screening due to its eligibility criteria.

In this study, we explored whether demographic characteristics are associated with being assessed ineligible for medication abortion with testing and ineligible without testing when applying the no-test protocol criteria, as well the proportion ineligible without testing when applying no-test criteria yet eligible with testing (false positives).

## Methods

### Study design and procedures

Here we present a secondary analysis of data collected as part of a study designed to assess the accuracy of abortion patients’ eligibility for medication abortion as determined using their self-reported health history as compared with clinician assessment with ultrasound and other testing. From June 2021 to December 2022, we recruited people seeking abortion from nine facilities in eight U.S. states (Arizona, California, Florida, Illinois, Iowa, North Dakota, Pennsylvania, and Tennessee). All study facilities required an ultrasound prior to abortion and offered both medication and procedural abortion. To be eligible, participants had to be seeking medication or procedural abortion, ages 15 and older (≥18 at select sites), able to speak and read English or Spanish, and without a prior ultrasound for the current pregnancy at the facility. Before ultrasound or clinician contact, patients completed a brief electronic eligibility survey, and those who consented and were eligible were directed to complete an electronic survey in English or Spanish. Patients’ gestational duration and type of abortion they sought were not included in the eligibility criteria. The survey asked participants to indicate their pregnancy duration, pregnancy symptoms, and medical history related to their eligibility for medication abortion, as well as other demographic and background characteristics. After the abortion visit, clinicians completed a standardized form where they indicated patients’ eligibility for medication abortion with ultrasound and other testing and reasons for ineligibility. Patient and clinician data were not shared with each other. We remunerated participants with a $5 gift card for completing the eligibility survey and an additional $25 gift card for completing the survey. The study received ethical approval from the University of California, San Francisco Institutional Review Board.

### Outcome measures

While facilities had slight differences in medication abortion eligibility criteria (gestational duration threshold ranging from 70 to 77 days, whether anemia was a contraindication, and use of medication abortion in cases of pregnancy of unknown location), we utilized the mifepristone label, American College of Obstetricians and Gynecologists (ACOG), and National Abortion Federation (NAF) practice guidance and set a gestational duration threshold of >77days, which is consistent with NAF guidelines and provider practice, as criteria to determine ineligibility for medication abortion.^1,26–30^ We measured three ineligibility outcomes: (1) ineligible with testing including ultrasound and any other indicated pre-abortion screening tests, (2) ineligible for no-test medication abortion when applying criteria in a no-test screening protocol, and (3) ineligible according to no-test screening yet eligible with testing (false positives), meaning they were incorrectly classified as “positive” for ineligibility using the no-test screening approach.

#### Ineligibility with testing

We considered clinician-assessed ineligibility with testing as an estimate of the true proportion of patients ineligible for medication abortion. Clinicians relied on information from the ultrasound and other pre-abortion screening tests (pregnancy and hemoglobin [Hgb]/hematocrit tests as indicated) to indicate whether patients had any medical reasons for ineligibility for medication abortion, by recording the following on a standardized form: (a) gestational duration >77 days; (b) concern for possible ectopic pregnancy; (c) Hgb <10.0 g/dL or clinical diagnosis of severe anemia; (d) other contraindication; and (e) other reason, with a space to specify. Contraindications included IUD in place, chronic adrenal failure, hemorrhagic disorder, concurrent anticoagulant therapy, inherited porphyria, concurrent long term-corticosteroid use, or allergy to mifepristone, misoprostol, or other prostaglandins. We conducted follow-up chart reviews for cases with key missing data (e.g., gestational duration and reason for ineligibility), inconsistent responses, or ectopic pregnancy concerns (e.g., without confirmed intrauterine pregnancy), to fill in or resolve inconsistencies.

#### No-test ineligibility

We utilized 13 eligibility criteria as reported and worded in a published sample no-test protocol.^1^ Six of the criteria relied on patient survey responses and seven relied on clinicians’ responses recorded on a standardized form. The patient survey criteria included: (1) *pregnancy duration* >77 days based on three items (a) “Using the calendar, please select the first day of your last menstrual period (LMP). If you know the approximate day, please select that date,” (b) “Select the date you think you got pregnant. If you are unsure but can estimate a date, please select that date,” and (c) “How many weeks pregnant do you think you are today?” We coded gestational duration >77 days on any of the three questions or missing or don’t know on all three questions as ineligible. We utilized this composite measure because it has been shown to have similar accuracy yet more sensitivity than a single measure^31^; (2) *suspected ectopic pregnancy* was based on four items (“had moderate or severe pain in the lower part of your belly in the past week,” “had vaginal bleeding or spotting in the past week,” “ever had or currently have an ectopic pregnancy,” and “ever had surgery on your fallopian tubes or had your tubes tied”; and (3) *anemia* (“told by a doctor or health care provider that you currently have a low red blood cell count, also known as anemia”). Conservatively, we coded all “yes” and “not sure” responses as ineligible. The remaining seven criteria relied on clinician-reported reasons for ineligibility since with the no-test protocol clinicians interact with the patient to confirm their medical history. These included IUD in place, chronic adrenal failure, hemorrhagic disorder, concurrent anticoagulant therapy, inherited porphyria, concurrent long-term corticosteroid use, and allergy to mifepristone, misoprostol, or other prostaglandins. We also conducted a sensitivity analysis applying a less strict no-test screening approach by removing patient-reported pain, bleeding/spotting, and anemia as ineligibility criteria.

#### False positives

We estimated the proportion incorrectly assessed as ineligible for medication abortion using no-test screening by subtracting the number ineligible with testing from the number ineligible without testing, divided by the full sample.

### Statistical analysis

To assess whether structurally oppressed communities are disproportionately ineligible according to each approach, we selected covariates based on *a priori* assumptions of the population characteristics that are associated with lacking access to high quality nonjudgmental health care, later pregnancy discovery, and having other contraindications to medication abortion.^21–23,25^ Demographic covariates included age, race/ethnicity, parity, lives in a household that speaks a language other than English, and lives in a household that experienced food or housing insecurity in the past year. Food or housing insecurity was measured by combining responses to three questions where we asked participants whether in the past 12 months they or others in their household (1) had ever cut the size of meals or skipped meals because there wasn’t enough money for food; (2) were worried food would run out before they had money to buy more; and (3) were not able to pay for their mortgage, rent, or utility bills. We coded “yes,” “don’t know,” “sometimes true,” and “often true” responses as food or housing insecure.

We conducted descriptive statistics to summarize participants’ sociodemographic background characteristics, proportion ineligible according to our three primary outcomes and reasons for ineligibility. We conducted bivariable and multivariable mixed-effects logistic regression to model each outcome and explore the characteristics associated with the five most common patient-reported reasons for ineligibility: (1) bleeding symptoms, (2) pain symptoms, (3) anemia, (4) pregnancy duration >77 days, and (5) has had or currently has ectopic pregnancy. We estimated the marginal mean proportion based on the post-estimated multivariate results. For variables with two or more categories (race/ethnicity and age-group), statistical significance was based on binary contrasts where each group was compared with the overall proportion, to avoid centering an arbitrary group. We used mixed-effects regression to account for clustering by recruitment site, used case-wise deletion methods for missing covariate data, which was low (0.4–1.8% missing), performed analyses in Stata version 18, and set statistical significance at *p* ≤ 0.05.

## Results

Among the 2,846 people approached, 1,775 (62%) were interested in the study, 1,591 were eligible, and 1,438 consented to participate and had complete clinician data. We excluded 52 people with incomplete survey data, leaving a final analytical sample of 1,386 participants. Most participants completed the survey in English (99%, 1,377/1,386). Participant characteristics are presented in Table 1. Over three-quarters (77.9%) of participants were eligible for medication abortion with testing; one in five (22.1%) were ineligible with testing, 71.5% were ineligible for no-test medication abortion, and 51.4% were classified as ineligible for no-test medication abortion yet eligible with testing (false positives; Table 2). When we removed patient-reported pain, bleeding/spotting, and anemia from no-test screening criteria as a sensitivity analysis, the proportion ineligible (29.7%) and false positives (13.6%) decreased considerably (Table 3).

**Table 1.** Characteristics of Participants Presenting for Abortion Care at Nine Abortion Facilities in Eight U.S. States from June 2021 to December 2022

	No.	%
Total	1,386	100
Age-group, years		
15–19	121	8.7
20–24	469	33.8
25–29	390	28.1
30–34	241	17.2
35–46	165	11.9
Parity		
Nulliparous	583	42.1
Parous	784	56.6
Missing	19	1.4
Gestational duration based on ultrasound		
<63 days	880	63.5
63–69 days	121	8.7
70–77 days	96	6.9
78–85 days	59	4.3
86–93 days	52	3.8
>93 days	116	8.4
Missing	62	4.5
Race and Hispanic ethnicity		
Black (non-Hispanic)	450	32.5
Hispanic/Latine	400	28.9
White (non-Hispanic)	398	28.7
Asian/Pacific Islander (non-Hispanic)	26	1.9
More than one race or other race (non-Hispanic)	102	7.4
Missing	10	0.7
Speaks language other than English at home		
Yes	217	15.7
No	1,162	83.8
Missing	7	0.2
Household experienced food or housing insecurity, past year		
Yes	690	49.8
No	693	50.0
Missing	3	0.2
Eligible for medication abortion according to assessment with testing		
Eligible	1,080	77.9
Ineligible	306	22.1
Eligible for medication abortion according to no-test screening		
Eligible	395	28.5
Ineligible	991	71.5

**Table 2.** Eligibility for Medication Abortion According to Assessment with and Without Testing and Proportion Ineligible by No-Test yet Eligible with Testing (False Positives) by Characteristics of Participants Presenting for Abortion Care at Nine Abortion Facilities in Eight U.S. States from June 2021 to December 2022 (*N* = 1,386)

	Eligibility according to assessment with testing^a^				Ineligible according to no-test screening^b^			Ineligible with no-test, yet eligible with testing		
	Eligible No. (Row %)	Ineligible No. (Row %)	Un-adjusted *p*	Adjusted *p*	Ineligible No. (Row %)	Un-adjusted *p*	Adjusted *p*	False positives No. (Row %)	Un-adjusted	Adjusted
Total ineligible	1,080 (77.9)	306 (22.1)			991 (71.5)			713 (51.4)		
Age-group, years										
15–19	88 (72.7)	33 (27.3)	0.20	**0.04**	105 (86.8)	**<0.001**	**0.001**	73 (60.3)	**<0.01**	0.14
20–24	373 (79.5)	96 (20.5)	0.15	0.22	353 (75.3)	0.21	0.27	263 (56.1)	**0.01**	**0.03**
25–29	305 (78.2)	85 (21.8)	0.64	0.35	281 (72.1)	0.90	0.94	204 (52.3)	0.42	0.25
30–34	191 (79.3)	50 (20.7)	0.30	0.14	163 (67.6)	0.73	0.13	117 (48.5)	0.58	0.94
35–46	123 (74.5)	42 (25.5)	0.35	0.59	89 (53.9)	**<0.001**	**<0.001**	56 (33.9)	**<0.001**	**<0.001**
Parity										
Parous	597 (76.1)	187 (23.9)	0.06	0.09	533 (68.0)	**<0.01**	0.31	369 (47.1)	**<0.001**	0.06
Nulliparous (reference)	468 (80.3)	115 (19.7)			443 (76.0)			333 (57.1)		
Missing	15 (78.9)	4 (21.1)	—	—	15 (78.9)	—	—	11 (57.9)	—	—
Race and Hispanic ethnicity										
Black (non-Hispanic)	327 (72.7)	123 (27.3)	0.07	0.17	317 (70.4)	0.46	0.51	212 (47.1)	0.07	0.17
Hispanic/Latine	320 (80.0)	80 (20.0)	0.99	0.18	303 (75.8)	0.77	0.73	226 (56.5)	0.86	0.37
White (non-Hispanic)	324 (81.4)	74 (18.6)	0.81	0.77	266 (66.8)	0.10	0.09	198 (49.7)	0.13	0.25
Asian/Pacific Islander (non-Hispanic)	20 (76.9)	6 (23.1)	0.79	0.94	19 (73.1)	0.73	0.69	14 (53.8)	0.86	0.56
More than one race or other race (non-Hispanic)	80 (78.4)	22 (21.6)	0.53	0.93	78 (76.5)	0.48	0.51	56 (54.9)	0.92	0.90
Missing	9 (90.0)	1 (10.0)	0.39	—	8 (80.0)	0.61	—	7 (70.0)	0.30	—
Speaks language other than English at home										
Yes	168 (77.4)	49 (22.6)	0.91	0.35	163 (75.1)	0.42	0.64	118 (54.4)	0.41	0.36
No (reference)	906 (78.0)	256 (22.0)			821 (70.7)			589 (50.7)		
Missing	6 (85.7)	1 (14.3)	—	—	7 (100)	—	—	6 (85.7)	—	—
Household experienced food or housing insecurity, past year										
Yes	522 (75.3)	171 (24.7)	**0.01**	**0.02**	525 (75.8)	**<0.001**	**<0.001**	372 (53.7)	0.11	0.10
No (reference)	555 (80.4)	135 (19.6)			464 (67.2)			339 (49.1)		
Missing	3 (100.0)	0 (0.0)	—	—	2 (66.7)	—	—	2 (66.7)	—	—

*p*-Value based on mixed-effects bivariable and multivariable logistic regression using case-wise deletion methods where missing cases are removed. For variables with two or more categories (race/ethnicity and age-group), statistical significance is based on binary contrasts where each group is compared with the balanced grand mean; **B**old**** text indicates statistical significance at *p* < 0.05.

^a^ Reasons for ineligibility according to assessment with testing included clinician assessed pregnancy duration >77 days, severe anemia, concern for ectopic pregnancy, negative pregnancy test, suspected miscarriage or pregnancy loss, IUD in place, chronic adrenal failure, hemorrhagic disorder, concurrent anticoagulant therapy, inherited porphyria, concurrent long-term corticosteroid use, and allergy to mifepristone or misoprostol.

^b^ Reasons for ineligibility according to no-test protocol included patient-reported pregnancy duration >77 days, bleeding symptoms, pain symptoms, severe anemia, and concern for ectopic pregnancy and clinician assessed IUD in place, chronic adrenal failure, hemorrhagic disorder, concurrent anticoagulant therapy, inherited porphyria, concurrent long-term corticosteroid use, and allergy to mifepristone or misoprostol.

IUD, intrauterine device.

**Table 3.** Reasons for Ineligibility for Medication Abortion by Screening Approach Among People Presenting for Abortion Care at Nine Abortion Facilities in Eight U.S. States from June 2021 to December 2022 (*N* = 1,386)

	Ineligible according to assessment with testing^a^		Ineligible according to no-test protocol^b^		Screened ineligible with no-test, yet eligible with testing (false positives)	
	No.	Row %	No.	Row %	No.	Row %
Total	306	100%	991	100%	713	100%
Reasons for ineligibility (patient survey/clinician assessment)	** **		** **	** **		** **
Pregnancy duration >77 days or not sure *(patient survey)*/pregnancy duration according to ultrasound *(clinician assessment)*	227	74.2%	348	35.1%	140	19.6%
Concern for ectopic						
Had moderate or severe pain in the lower part of your belly, past week or not sure *(patient survey)*	n/a		614	62.0%	489	68.6%
Had vaginal bleeding or spotting in the past week or not sure *(patient survey)*	n/a		207	20.9%	150	21.0%
Ever had or currently has an ectopic pregnancy or not sure *(patient survey)*/Concern for possible ectopic (*clinician assessment)*	12	3.9%	122	12.3%	92	12.9%
Ever had tubal surgery or tubes tied or not sure *(patient survey)*	n/a		15	1.5%	7	1.0%
IUD in place *(clinician assessment)*	3	1.0%	3	1.2%	0	0.0%
Negative pregnancy test at the clinic *(clinician assessment)*	20	6.5%	n/a		n/a	
Suspected miscarriage or early pregnancy loss *(clinician assessment)*	15	4.9%	n/a		n/a	
Medical contraindications						
Ever told by doctor has low blood count or anemia/not sure *(patient survey)*/Hemoglobin value <10.0 or clinician documented severe anemia *(clinician assessment)*	35	11.4%	277	28.0%	204	28.6%
Chronic adrenal failure *(clinician assessment)*	0	0.0%	0	0.0%	0	0.0%
Hemorrhagic disorder *(clinician assessment)*	2	0.7%	2	0.2%	0	0.0%
Concurrent anticoagulant therapy *(clinician assessment)*	3	1.0%	3	1.2%	0	0.0%
Inherited porphyria *(clinician assessment)*	0	0.0%	0	0.0%	0	0.0%
Concurrent long-term corticosteroid use *(clinician assessment)*	1	0.4%	1	0.1%	0	0.0%
Allergy to mifepristone, misoprostol, or other prostaglandins *(clinician assessment)*	0	0.0%	0	0.0%	0	0.0%
Sensitivity analysis (*N* = 1,386)						
Removing pain, bleeding and anemia *(patient survey)*	n/a		412	29.7%	189	13.6%

^a^ Reasons for ineligibility according to assessment with testing included clinician-reported pregnancy duration >77 days, severe anemia, concern for ectopic pregnancy, negative pregnancy test, suspected miscarriage or pregnancy loss, IUD in place, chronic adrenal failure, hemorrhagic disorder, concurrent anticoagulant therapy, inherited porphyria, concurrent long-term corticosteroid use, and allergy to mifepristone or misoprostol.

^b^ Reasons for ineligibility according to no-test protocol criteria included patient-reported pregnancy duration >77 days, bleeding symptoms, pain symptoms, severe anemia, and concern for ectopic pregnancy and clinician assessed IUD in place, chronic adrenal failure, hemorrhagic disorder, concurrent anticoagulant therapy, inherited porphyria, concurrent long-term corticosteroid use, and allergy to mifepristone or misoprostol; sensitivity analyses included all of above but removing pain, bleeding, and anemia.

### Characteristics associated with ineligibility for medication abortion

When compared with the overall sample, people ages 15–19 were significantly more likely to be ineligible with testing in adjusted analyses (27.3% vs 22.1%, *p* = 0.04) but not unadjusted (*p* = 0.20) analyses, to screen ineligible for no-test medication abortion (86.8% vs 71.5%; unadjusted: *p* < 0.001; adjusted: *p* = 0.001), and to screen ineligible using the no-test protocol yet eligible with testing (false positives, 60.3% vs 51.4%; unadjusted *p* < 0.01; adjusted *p* = 0.14; Table 2). People living in households that experienced food or housing insecurity in the past year were significantly more likely to be ineligible with testing (24.7% vs. 19.6%; unadjusted *p* = 0.01; adjusted *p* = 0.02) and without testing (75.8% vs. 67.2%; unadjusted *p* < 0.001; adjusted *p* < 0.001), although they were not more likely to screen as false positives. People who were nulliparous were significantly more likely to screen ineligible using the no-test protocol (76.0% vs. 68.0%, *p* < 0.01) and to screen false positive (ineligible using the no-test protocol yet eligible with testing, 57.1% vs. 47.1%, *p* < 0.001) in unadjusted but not adjusted analyses.

### Characteristics associated with reasons for ineligibility

Among the 22.1% (*n* = 306) ineligible with testing, the most common clinician-reported reasons for ineligibility included pregnancy duration >77 days (74%), severe anemia (11%), and possible ectopic pregnancy (3.9%; Table 3). Among the 71.5% (*n* = 991) ineligible based on the no-test protocol, the most common patient-reported reasons for ineligibility included moderate/severe pelvic pain (62%), pregnancy duration >77 days (35%), concerns about anemia (28%), vaginal bleeding or spotting (21%), and history of or suspected ectopic pregnancy (12%). Similarly, the most common reasons for ineligibility without testing among the 51.4% (*n* = 713) who were determined as incorrectly screened as ineligible using the no-test protocol (false positives) included moderate or severe pelvic pain (69%), anemia (29%), and vaginal bleeding or spotting in the past week (21%).

In adjusted analyses, we examined the characteristics associated with the five most common patient-reported reasons for ineligibility among the patients determined ineligible applying no-test screening criteria. When compared with the overall sample, people ages 15–19 were significantly more likely to report pain symptoms (71.4% vs. 62.1%, *p* = 0.04), pregnancy duration >77 days (46.8% vs. 34.5%, *p* = 0.02), and less likley to report vaginal bleeding or spotting in the past week (13.4% vs. 20.9%, *p* = 0.02). People who were nulliparous were significantly more likely to report pain symptoms (70.3% vs. 55.6%, *p* < 0.001) and less likely to report pregnancy duration >77 days (29.7% vs. 38.6%, *p* = 0.01) and possible anemia (22.0% vs. 32.4%, *p* < 0.01) compared with parous participants. People living in a household that experienced food or housing insecurity in the past year were significantly more likely to report pain symptoms (66.3% vs. 57.4%, *p* < 0.01; Table 4).

**Table 4.** Among Those Assessed Ineligible According to No-Test Screening Criteria, the Most Common Reasons for Ineligibility by Participant Characteristics Based on Post-Estimate Results of Multivariable Mixed-Effects Logistic Regression Analyses (*N* = 972) Among People Presenting for Abortion Care at Nine Abortion Facilities in Eight U.S. States in the United States from June 2021 to December 2022

	Moderate/severe abdominal pain, past week		Pregnancy duration >77 days		Ever told by doctor has low blood count or anemia		Vaginal bleeding/spotting past week		Ever had or currently has ectopic pregnancy	
	Marginal mean^a^	*p*-Value	Marginal mean	*p*-Value	Marginal mean	*p*-Value	Marginal mean	*p*-Value	Marginal mean	*p*-Value
Total	62.1%		34.5%		27.7%		20.9%		12.4%	
Age-group^b^										
15–19	**71.4%**	**0.04**	**46.8%**	**0.02**	31.5%	0.37	**13.4%**	**0.02**	13.2%	0.94
20–24	65.3%	0.21	32.6%	0.21	31.2%	0.16	20.9%	0.86	11.2%	0.33
25–29	58.8%	0.29	33.1%	0.31	23.8%	0.14	19.4%	0.45	13.2%	0.92
30–34	61.5%	0.91	34.1%	0.60	25.3%	0.47	21.5%	0.94	12.0%	0.70
35–46	**51.5%**	**0.02**	33.7%	0.60	26.8%	0.84	**34.8%**	**0.001**	15.6%	0.42
Nulliparous										
No (reference)	55.6%		38.6%		32.4%		18.9%		10.5%	
Yes	**70.3%**	**<0.001**	**29.7%**	**0.01**	**22.0%**	**<0.01**	23.6%	0.12	14.8%	0.09
Race and Hispanic ethnicity^b^										
Black (non-Hispanic)	58.6%	0.11	34.8%	0.51	29.1%	0.74	20.8%	1.00	11.6%	0.98
Hispanic/Latine	65.2%	0.78	33.1%	0.26	31.4%	0.33	23.8%	0.34	11.4%	0.96
White (non-Hispanic)	59.9%	0.23	32.3%	0.17	**21.4%**	**0.04**	19.4%	0.63	15.3%	0.15
All other (non-Hispanic)	64.5%	0.98	42.2%	0.58	30.0%	0.82	28.4%	0.32	10.1%	0.81
More than one race (non-Hispanic)	72.2%	0.10	44.2%	0.17	28.9%	0.85	13.8%	0.08	9.9%	0.61
Speaks language other than English at home										
No (reference)	61.3%		34.1%		29.4%		20.8%		12.6%	
Yes	66.5%	0.28	36.6%	0.59	**19.8%**	**0.02**	21.2%	0.93	11.5%	0.75
Household has been food or housing insecure, past year										
No (reference)	57.4%		32.0%		27.0%		19.0%		11.7%	
Yes	**66.3%**	**<0.01**	36.8%	0.11	28.2%	0.68	22.7%	0.17	13.1%	0.51

^a^ Marginal mean is based on post-estimated results following multivariable logistic regression analyses adjusting for age, parity, race/ethnicity, language spoken at home, and experiences of food and housing insecurity.

^b^ For variables with two or more categories (race/ethnicity and age group) statistical significance is based on binary contrasts where each group is compared with the balanced grand mean.

**Bold** text indicates statistical significance at *p* < 0.05.

In adjusted analyses we examined the characteristics associated with the five most common patient-reported reasons for ineligibility among participants screened as ineligible using the no-test protocol yet eligible with testing (false positives): people who were ages 15–19 (79.8% vs. 68.8%, *p* = 0.04) and nulliparous (75.8% vs. 63.0%, *p* = 0.001) were more likely and people ages 35–46 (57.5% vs. 68.8%, *p* = 0.04) less likely to report pain symptoms; people who were nulliparous (21.9% vs. 33.9%, *p* < 0.01) and who spoke a language other than English at home (20.1% vs. 29.9%, *p* = 0.05) were significantly less likely than their counterparts to report possible anemia (Table 5).

**Table 5.** Among False Positives, the Most Common Reasons for Ineligibility by Participant Characteristics Based on Post-Estimate Results of Multivariable Mixed-Effects Logistic Regression Analyses Among People Presenting for Abortion Care at Nine Abortion Facilities in Eight U.S. States from June 2021 to December 2022 (*N* = 698)

	Moderate/severe abdominal pain, past week		Pregnancy duration >77 days		Ever told by doctor has low blood count or anemia		Vaginal bleeding/spotting past week		Ever had or currently has ectopic pregnancy	
	Marginal mean^a^	*p*-Value	Marginal mean	*p*-Value	Marginal mean	*p*-Value	Marginal mean	*p*-Value	Marginal mean	*p*-Value
Total	**68.8%**		**19.9%**		**28.2%**		**21.8%**		**13.0%**	
Age-group^b^										
15–19	**79.8%**	**0.04**	24.2%	0.44	28.4%	0.96	15.3%	0.15	12.4%	0.78
20–24	72.5%	0.24	20.0%	0.82	31.3%	0.30	22.4%	0.77	12.0%	0.53
25–29	65.6%	0.34	18.7%	0.49	24.9%	0.29	21.2%	0.91	13.5%	0.96
30–34	66.2%	0.52	18.3%	0.49	26.8%	0.73	23.2%	0.66	14.4%	0.76
35–46	**57.5%**	**0.04**	22.4%	0.71	29.6%	0.77	27.0%	0.28	14.9%	0.72
Nulliparous	** **				** **				** **	** **
No (reference)	63.0%		22.2%		33.9%		19.7%		10.6%	
Yes	**75.8%**	**0.001**	17.5%	0.18	**21.9%**	**<0.01**	24.2%	0.22	15.9%	0.07
Race and Hispanic ethnicity^b^										
Black (non-Hispanic)	65.0%	0.14	20.3%	0.55	26.9%	0.57	21.0%	0.84	13.5%	0.47
Hispanic/Latine	69.3%	0.71	19.3%	0.37	31.5%	0.55	22.9%	0.75	11.8%	0.85
White (non-Hispanic)	69.9%	0.82	17.5%	0.15	25.6%	0.37	22.5%	0.83	15.1%	0.23
All other (non-Hispanic)	72.3%	0.88	29.1%	0.46	31.7%	0.79	26.2%	0.61	7.0%	0.54
More than one race (non-Hispanic)	76.9%	0.27	27.3%	0.34	29.8%	0.89	16.7%	0.32	10.3%	0.82
Speaks language other than English at home									** **	** **
No (reference)	67.6%		19.9%		29.9%		21.0%		13.3%	
Yes	75.0%	0.41	20.2%	0.95	**20.1%**	**0.05**	25.3%	0.38	11.7%	0.70
Household has been food or housing insecure, past year										
No (reference)	64.8%		19.9%		26.0%		19.3%		12.5%	
Yes	72.3%	0.25	19.9%	1.00	30.3%	0.21	24.1%	0.13	13.5%	0.71

^a^ Marginal mean is based on post-estimated results following multivariable logistic regression analyses adjusting for age, parity, race/ethnicity, language spoken at home, and experiences of food and housing insecurity.

^b^ For variables with two or more categories (race/ethnicity and age group) statistical significance is based on binary contrasts where each group is compared with the balanced grand mean; bold text indicates statistical significance at *p* < 0.05.

## Discussion

Overall, we find that a large proportion of people—over half—who are likely eligible for medication abortion may be excluded from no-test medication abortion due to no-test screening eligibility criteria. Although about one in five people were ineligible for medication abortion according to assessment with testing, when we applied the no-test screening criteria, the proportion ineligible grew to over 70% and disparities widened. This proportion is somewhat larger than reported in a recent study restricted to adults seeking medication abortion which applied fewer eligibility criteria in their self-administered screening approach: LMP >70 days or uncertain LMP, history of ectopic pregnancy, IUD in place within past 2 months, and irregular menses.^32^ However, that study was limited in that it did not explore whether these screening criteria disproportionately excluded certain population groups.

Both assessment with and without testing resulted in disproportionate exclusion of certain populations in particular young people and people living in households that had experienced food or housing insecurity. This may be due in part to later pregnancy recognition among people who lack access to home pregnancy tests which can lead to later presentation to care. This finding is consistent with other research showing that young people, people who were nulliparous, and who experienced food insecurity were significantly more likely to confirm pregnancy after 6 weeks, often due to lack of access of home pregnancy tests, when compared with their counterparts.^25^

Although ACOG recommends that people suspected of anemia have their Hgb or hematocrit assessed before treating with medication abortion because of concerns about heavy bleeding in patients with anemia, ACOG also points to the lack of studies examining the risks of using medication abortion among anemic patients.^27^ In the current study, over one-quarter of participants who were screened ineligible for no-test medication abortion yet eligible with testing reported possible anemia, in particular people who were parous. Future research should examine the appropriateness of including anemia as a contraindication and/or develop more specific screening questions, as it may disproportionately exclude many people who are in fact eligible for medication abortion.

ACOG and the no-test protocol recommend that patients with bleeding and pelvic pain symptoms be clinically evaluated for ectopic pregnancy before treatment.^1,27,33^ Patient-reported pain symptoms in the past week—moderate or severe pain in lower belly—were the most common patient-reported reason for ineligibility, reported by 62% of those ineligible for medication abortion according to the no-test criteria and 69% of people who were incorrectly screened as ineligible (false positives). Young people, people who were nulliparous, and people who experienced food or housing insecurity were more likely to report pelvic pain symptoms, leading them to disproportionately screen ineligible for no-test medication abortion. Self-reported bleeding symptoms were also common and reported by over one in five patients who were incorrectly screened as ineligible (false positives). Our subjective pain and bleeding measures may have been difficult to interpret, in particular for people who lack pregnancy experience as a reference point. In an analysis of these same data, after testing, clinicians reported concern about possible ectopic pregnancy in just 12 participants, of whom only five reported pain symptoms.^34^ Future work should focus on finding better ways to ensure people with possible ectopics get appropriate and timely care. This may include developing new screening questions that are more accurate at identifying ectopic pregnancies. We also must consider whether it may be more appropriate to focus on ensuring appropriate monitoring and evaluation of symptoms for people with suspected ectopics after treatment rather than excluding them from no-test care.

## Limitations

This study has important limitations. Our sample was limited to people accessing abortion at facilities and thus not generalizable to those who cannot access abortion in-person and for whom no-test screening may serve an unmet need for care. Future research should examine the impact of applying no-test screening criteria among people seeking telehealth abortion care, among people who self-manage their abortions, and among patients who desire but are unable to access abortion care. Furthermore, while our application of ineligibility criteria used in the no-test protocol mimicked a real-world setting where a clinician interacts with patients—whether in person or via telephone, video, or text—this approach may have overestimated ineligibility. In reality, clinicians would likely ask follow-up questions in response to patients’ answers, reducing estimates of ineligibility. Moreover, our conservative assumption that all “not sure” responses were indicators of ineligibility may have further inflated our ineligibility estimates. Finally, our analyses among subsamples of patients assessed ineligible may have been underpowered to detect true differences by participant characteristics. Future research should examine the characteristics of people inappropriately excluded when applying the no-test screening criteria and refine protocols in order to better understand and reduce any inequities that may be introduced through various screening approaches.

## Conclusions

No-test screening has been critical to serving patients who otherwise would not have access to abortion care, by enabling providers to reach people in every U.S. state, including states that ban abortion. However, we find that the no-test protocol may not be reaching the most marginalized and structurally oppressed populations. When applying a conservative screening approach, no-test screening may inadvertently exclude many patients who are eligible for medication abortion, potentially exacerbating inequitable access to care, by limiting people’s options. People referred for in-person testing must face added costs, time, and travel, potentially leading some to forgo needed care. Furthermore, restrictions on abortion may further limit people from accessing their preferred type of abortion care. Policies that make it harder for providers to offer procedural abortion in an outpatient setting might make it so that only medication abortion is available to patients in some settings, or alternatively, Risk Evaluation and Mitigation Strategy (REMS) requirements might prevent providers from offering medication abortion, limiting people’s options in other settings to only procedural abortion. Our study findings call for a need to explore how people interpreted current screening questions, which were not sensitive to identifying only patients who truly face medical risks from using no-test medication abortion. More research is needed to understand why certain groups are more likely to be assessed as ineligible for no-test medication abortion, whether due to higher incidence of some contraindications, clinician bias, inadequate questions to measure pain and bleeding symptoms, pregnancy duration, and anemia, or other reasons. Ensuring equitable access to abortion care will require that all people have access to a range of safe and effective options, including no-test and in-person abortion care in order to best meet people’s health care needs.

## Abbreviations Used

ACOG: American College of Obstetricians and Gynecologists
IUD: Intrauterine Device
NAF: National Abortion Federation
REMS: Risk Evaluation Mitigation Strategy
